# Editorial: Effects of Combined EMF Exposures and Co-exposures

**DOI:** 10.3389/fpubh.2018.00230

**Published:** 2018-08-20

**Authors:** Mats-Olof Mattsson, Olga Zeni, Myrtill Simkó, Maria Rosaria Scarfì

**Affiliations:** ^1^SciProof International (Sweden), Östersund, Sweden; ^2^Consiglio Nazionale Delle Ricerche (CNR), Istituto per il Rilevamento Elettromagnetico dell'Ambiente (IREA), Naples, Italy

**Keywords:** electromagnetic field (EMF), static magnet fields, radiofrequency magnetic fields, low frequency magnetic fields, MRI-magnetic resonance imaging, radical homeostasis, cancer, heat response

Increasingly, assessment of health effects of environmental exposures is focusing on effects of combinations of agents [cf. (1)]. Electromagnetic fields (EMFs) that are considered here range from static fields up to microwave frequencies, and interact with living matter in different ways across the spectrum. However, few studies have investigated effects of either combinations of different EMFs or effects of co-exposures where EMF is one of the agents (2). According to a study based on text mining, at most 5–7% of EMF studies related to health are concerned with combination-type effects (3).

The papers in the Topic cover several parts of the EMF spectrum and different levels of biological organization. The papers by Frankel et al. and by Sannino et al. deal with the complex exposure that exists in magnetic resonance imaging (MRI) suites. This diagnostic tool is a prime example of a device, which is emitting in different parts of the EMF spectrum. There is a static magnetic field, which is permanently present, and switch-gradient low frequency fields (kHz) and radiofrequency (RF) fields that are used during the investigation. Frankel and co-authors provide exposure data and point to that exposure assessment for personnel working in MRI suites is very difficult. Not only since different instruments have different settings and are used for different investigations, but also that the exposure to the different EMFs can be combined with possible chemical (contrast agents) exposure and even ionizing radiation (PET scans). The research paper by Sannino et al. deals with exposure assessment and biomonitoring of MRI workers. The subjects were either MRI workers belonging to different professional categories, or hospital personnel not subjected to the MRI environment (reference control group). The exposure to the MRI static magnetic fields, measured by personal monitoring, showed compliance with directives for occupational exposure, although high day-to-day and worker-to-worker exposure variability was recorded. An easy-to-use application tool for rapid and qualitative evaluation of motion-induced, time-varying electric field exposure was developed in the study. Regarding the biomonitoring, both spontaneous and mitomycin C—induced genotoxicity was evaluated by a cytokinesis block micronucleus assay in lymphocytes from MRI workers and reference control workers. No statistically significant difference between the two groups was seen, although an increase in the number of subjects is needed to gain robust statistics and reliable results.

A review by Sienkiewicz et al. has its origin in the possible discrepancy between epidemiological studies on mobile phone use and cancer incidence, and the corresponding animal studies (2). The signals that are investigated in these two types of studies differ significantly. The real-life exposure contains many frequencies, signals, and intensities. In contrast, most animal studies used a single 2G mobile phone-derived signal at one or very few intensities. The authors ask if this is the reason for outcome differences, and arrive at the conclusion that further animal studies using multiple frequencies will not provide an answer to the question. They argue that there is no convincing evidence regarding carcinogenicity from any of the two types of studies, and furthermore that there is no plausible interaction mechanism that suggests that two or more RF signals would produce anything more than additive effects, if any effects at all.

The remaining papers in the Research Topic are addressing extremely low frequency magnetic fields (ELF-MF, 50/60 Hz), and how these can influence the effect of other agents.

Zeni et al. have searched the available literature for studies that investigated cellular responses to both heat treatment and ELF-MF. ELF-MF alone has been shown to induce or increase gene expression of heat shock proteins (HSP), similar to heat treatment. The heat treatment causes different cellular responses if it is a mild heat stress (temperatures 39–43°C), which counteracts detrimental effects on existing proteins' structures and thus ensures normal cellular functions, and lethal heat stress (44–45°C), which ends with necrosis. In the former case, HSP expression is a key factor in the protection of cell integrity. Zeni et al. found that ELF-MF behaves as a mild stressor, inducing thermotolerance. When combined with mild heat stress, the effects of MF are potentiating the effects of heat. So far, available studies have not been able to indicate if heat and MF are operating by the same or different mechanism(s). The observed sensitivity to MF exposure seems not to be primarily due to exposure conditions (frequency, flux density, exposure duration), but more likely reflects the status of the biological material.

In the review by Falone et al., the authors asked if ELF-MF exposure can influence cancer drug resistance in tumor cells studied *in vitro*. The mechanism behind multidrug resistance is often unclear. However, evidence indicates that ROS formation and/or disturbed redox balance is involved. Since also many ELF-MF studies have focused on radical homeostasis, the question arises if there are specific radical-related effects of simultaneous or sequential exposures to ELF-MF and chemicals that act to influence the carcinogenic process. The authors have focused on treatments with differentiating agents, with cytostatic/cytotoxic drugs, with DNA-damaging challenges, and with conditions that promote anti-oxidative and/or detoxification processes. Although many of the results are contradictory, the authors found that ELF-MF can modify how tumor cells respond to either pro-differentiating or cytostatic/cytotoxic conditions.

Both Zeni et al. and Falone et al. suggest that the inconsistent outcomes of the studies are due to differences in the study designs and to insufficient control of experimental conditions.

The very complex exposure situation in real-life environments has been well illustrated in this Research Topic, and the need for better understanding of basic biological interaction mechanisms is obvious from the analyses presented here. The area is very much under-investigated, and the full impact and potential of EMF exposures for both possible adverse and beneficial effects cannot be realized without substantial additional research efforts.

## Author contributions

All authors listed have made a substantial, direct and intellectual contribution to the work, and approved it for publication.

### Conflict of interest statement

The authors declare that the research was conducted in the absence of any commercial or financial relationships that could be construed as a potential conflict of interest.
